# Supplementary material for: MetaRibo-Seq measures translation in microbiomes
Source: Nat Commun. 2020 Jun 29;11:3268. doi: 10.1038/s41467-020-17081-z (PMC7324362; doi:10.1038/s41467-020-17081-z)
Supplement: Supplementary file 10 — Supplementary Data 7 [file 41467_2020_17081_MOESM10_ESM.zip › File2/Confidence_High_Taxonomy/110119_out.krona.html]

Javascript must be enabled to view this page.

members
magnitude
magnitudeUnassigned
count
unassigned
taxon
rank

110119\_out

55

2
55
superkingdom

1239
55
phylum

55
class
186801

55
order
186802

family
54
186803

54
genus
841

species
1

SRS015579\_contig\_number\_25852
2292367

53
species

SRS013940\_contig\_number\_contig-100\_10538.103328SRS014613\_contig\_number\_20850SRS014855\_contig\_number\_14677SRS015190\_contig\_number\_contig-100\_1204.95507SRS015264\_contig\_number\_21766SRS015431\_contig\_number\_2334SRS015578\_contig\_number\_27029SRS015854\_contig\_number\_17999SRS015890\_contig\_number\_15752SRS015960\_contig\_number\_8749SRS017821\_contig\_number\_22703SRS018984\_contig\_number\_27777SRS019068\_contig\_number\_54157SRS019397\_contig\_number\_9850SRS019685\_contig\_number\_contig-100\_24859.44837SRS022137\_contig\_number\_contig-100\_857.111177SRS024435\_contig\_number\_12781SRS024549\_contig\_number\_4783SRS043001\_contig\_number\_10157SRS043411\_contig\_number\_5104SRS043701\_contig\_number\_contig-100\_219.114862SRS049900\_contig\_number\_5628SRS050941\_contig\_number\_5966SRS052027\_contig\_number\_14471SRS054956\_contig\_number\_contig-100\_208.93642SRS055017\_contig\_number\_29172SRS056259\_contig\_number\_2075SRS056273\_contig\_number\_31085SRS056519\_contig\_number\_18521SRS058070\_contig\_number\_10582SRS075341\_contig\_number\_13971SRS075716\_contig\_number\_340SRS077502\_contig\_number\_contig-100\_12747.12747SRS077552\_contig\_number\_18024SRS077641\_contig\_number\_5669SRS098061\_contig\_number\_11635SRS104084\_contig\_number\_13863SRS1041130\_contig\_number\_3575SRS1041144\_contig\_number\_contig-100\_23462.23463SRS104975\_contig\_number\_2438SRS1055022\_contig\_number\_5677SRS142503\_contig\_number\_49692SRS142781\_contig\_number\_11201SRS143070\_contig\_number\_15722SRS143598\_contig\_number\_23262SRS144714\_contig\_number\_18488SRS146812\_contig\_number\_18993SRS147919\_contig\_number\_2853SRS148091\_contig\_number\_10214SRS148159\_contig\_number\_43813SRS148784\_contig\_number\_20712SRS148817\_contig\_number\_contig-100\_308.309SRS893378\_contig\_number\_20176
166486

541000
1
family

216851
1
genus


SRS076756\_contig\_number\_contig-100\_1428.77061
853
species
1
